# Integrated Analysis of LncRNA-mRNA Co-Expression Profiles in Patients with Moyamoya Disease

**DOI:** 10.1038/srep42421

**Published:** 2017-02-08

**Authors:** Wen Wang, Faliang Gao, Zheng Zhao, Haoyuan Wang, Lu Zhang, Dong Zhang, Yan Zhang, Qing Lan, Jiangfei Wang, Jizong Zhao

**Affiliations:** 1 Department of Neurosurgery, Beijing Tiantan Hospital, Capital Medical University, Beijing, 100050, China; 2 Department of Neurosurgery, The Second Affiliated Hospital of Soochow University, Suzhou, 215123, China; 3 China National Clinical Research Center for Neurological Diseases, Beijing, 100050, China; 4 Beijing Key Laboratory of Translational Medicine for Cerebrovascular Diseases, Beijing, 100050, China; 5 Beijing Neurosurgical Institute, Capital Medical University, Beijing, 100050, China; 6 Department of Neurosurgery, Zhujiang Hospital, Southern Medical University, Guangzhou, 510280, China; 7 Department of Ophthalmology, School of Medicine, Shandong University, Jinan, 250012, China

## Abstract

Moyamoya disease (MMD) is an idiopathic disease associated with recurrent stroke. However, the pathogenesis of MMD remains unknown. Therefore, we performed long noncoding RNA (lncRNA) and messenger RNA (mRNA) expression profiles in blood samples from MMD patients (N = 15) and healthy controls (N = 10). A total of 880 differentially expressed lncRNAs (3649 probes) and 2624 differentially expressed mRNAs (2880 probes) were obtained from the microarrays of MMD patients and healthy controls (P < 0.05; Fold Change >2.0). Gene ontology (GO) and pathway analyses showed that upregulated mRNAs were enriched for inflammatory response, Toll-like receptor signaling pathway, chemokine signaling pathway and mitogen-activated protein kinase (MAPK) signaling pathway among others, while the downregulated mRNAs were enriched for neurological system process, digestion, drug metabolism, retinol metabolism and others. Our results showed that the integrated analysis of lncRNA-mRNA co-expression networks were linked to inflammatory response, Toll-like signaling pathway, cytokine-cytokine receptor interaction and MAPK signaling pathway. These findings may elucidate the pathogenesis of MMD, and the differentially expressed genes could provide clues to find key components in the MMD pathway.

Moyamoya disease (MMD) is a congenital disease that is characterized by stenosis of terminal internal carotid arteries and a hazy network of basal collaterals^1^. Recurrent stroke is common among MMD patients, and the standard treatment for MMD is revascularization surgery^2^,^3^. Although genome-wide and locus-specific association studies identified RNF213 as an important susceptibility gene of MMD^4^, few studies focus on the dysregulated genes and the pathogenesis of MMD still remains unknown.

Long noncoding RNAs (LncRNAs) are RNA molecules longer than 200 nucleotides without protein-coding ability^5^. Many studies have revealed a wide range of functional activities of lncRNAs^6^,^7^, including chromatin remodeling, transcriptional control and post-transcriptional processing. The dysregulation of lncRNAs might contribute to inflammatory response^8^, and several studies have reported that lncRNAs are associated with various inflammatory conditions^9^,^10^,^11^,^12^. TUG1 could decrease inflammation *in vivo*^13^, and ANRIL could regulate inflammatory responses through the NF-κB pathway^14^. JMJD1A and MALAT1 can reduce the activity of mitogen-activated protein kinase (MAPK) signaling in glioma cells and gastric cancer^15^,^16^, respectively. Genetic and environmental factors may play important roles in MMD development^17^. In a previous study, lncRNA expression profiles produced completely different clusters, and the MAPK signaling pathway was found to play a core role in this pathway network^18^. Co-expression analysis is widely used to elucidate the relationship between lncRNAs and messenger RNAs (mRNAs)^19^,^20^. It can elucidate the key lncRNAs and help to find a new regulation mechanism.

Understanding dysregulated lncRNAs and mRNAs is important for the diagnosis and treatment of patients with MMD. Therefore, we performed a microarray to examine lncRNA and mRNA expression profiles in blood samples from MMD patients and healthy controls. A total of 880 differentially expressed lncRNAs (3649 probes) and 2624 mRNAs (2880 probes) were identified. We further performed four co-expression networks in inflammatory response, the Toll-like signaling pathway, cytokine-cytokine receptor interaction and the MAPK signaling pathway. The integrated analysis of the differentially expressed lncRNAs and mRNAs may provide clues to find genes with active roles in pathogenesis of MMD.

## Results

### The clinical characteristics of included MMD patients

We included 15 MMD patients and 10 healthy controls in our study. The clinical characteristics of the included patients are shown in Table 1. There were 6 patients with intraventricular hemorrhage (IVH), 1 with subarachnoid hemorrhage (SAH), 3 with transient ischemic attack (TIA) and 5 with infarction as their initial clinical findings. Based on that, we grouped the patients into the hemorrhagic group (HG), the ischemic group (IG) and the ischemic and hemorrhagic group (IHG). The MMD and Control groups had similar age and sex distributions (Table 1).

### Identification of differentially expressed lncRNAs and mRNAs

**Figure 1:**
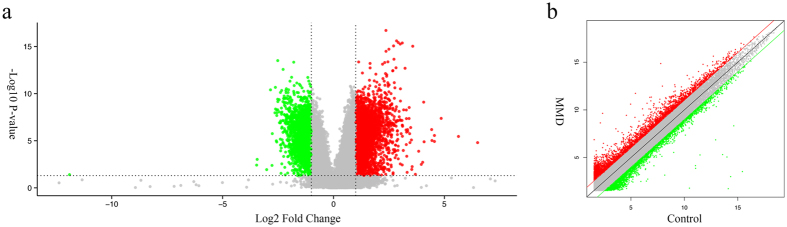
mRNA expression profile in MMD patients and healthy controls. (**a**) Volcano plots of mRNAs expression levels between MMD and control group. (**b**) Scatter plots of mRNAs expression levels between the MMD and control groups. The red dots represented upregulated mRNAs, and the green dots represented downregulated mRNAs (P < 0.05; Fold Change >2.0).

**Figure 2:**
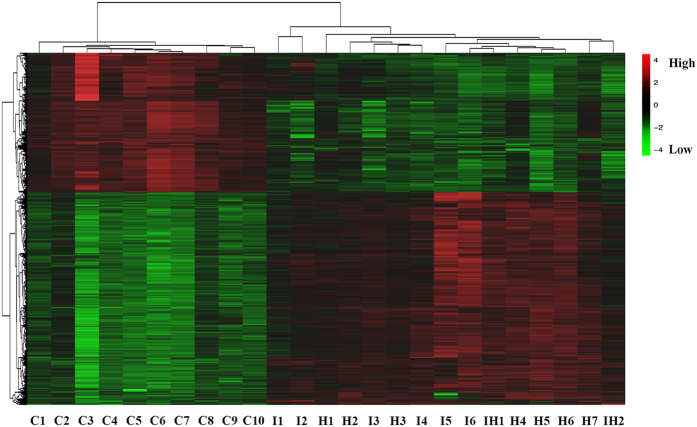
Heat map of differentially expressed mRNAs of MMD patients and healthy controls.

The list of lncRNAs and their expression profiles were extracted in our previous study^18^. In brief, we identified 880 differentially expressed lncRNAs (3649 probes) and 2624 mRNAs (2880 probes) from the microarrays of MMD patients and healthy controls (P < 0.05; Fold Change >2.0) (Supplementary Table S1). Of those, 1746 upregulated mRNAs and 878 downregulated mRNAs were identified. A volcano plot was created and scatter analyses were conducted to identify differences among mRNAs (Fig. 1a,b). We further created a heat map of differentially expressed mRNAs (Fig. 2).

**Figure 3:**
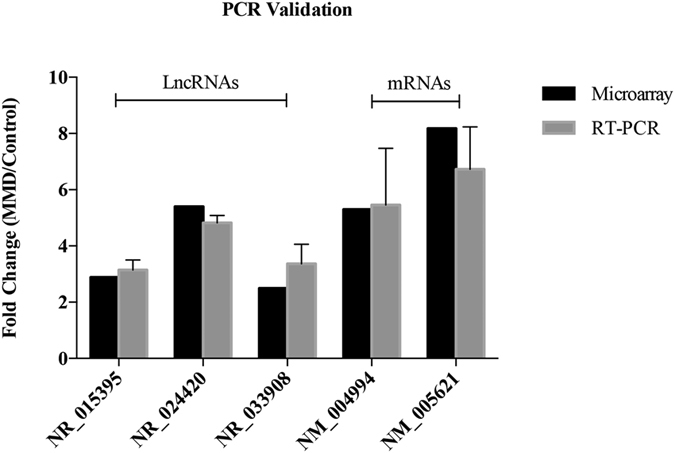
Validation of microarray results using quantitative real-time polymerase chain reaction (qRT-PCR).

We also selected several differentially expressed genes randomly and further performed quantitative real-time polymerase chain reaction (qRT-PCR) to examine their expression levels. The qRT-PCR results suggest that the fold changes observed in the microarray analysis were robust (Fig. 3).

### Examination of the function of differentially expressed mRNAs

**Figure 4:**
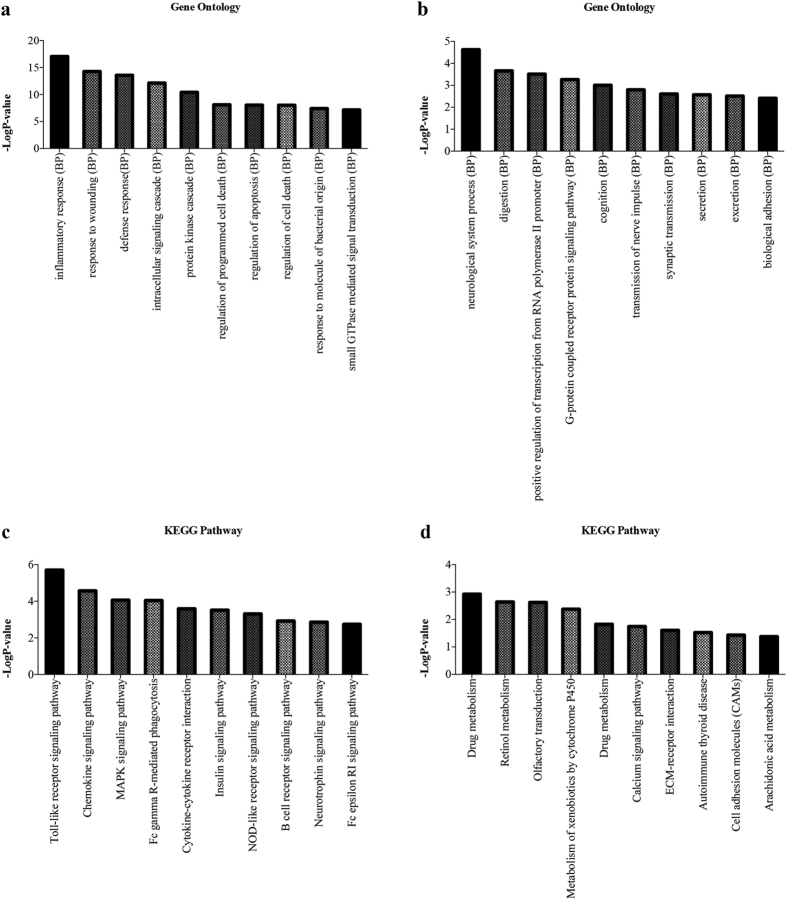
Gene ontology and KEGG Pathway analysis of 2624 differentially expressed mRNAs. (**a**) The top 10 GO terms upregulated in MMD patients compared with healthy controls. (**b**) The top 10 GO terms downregulated in MMD patients compared with healthy controls. (**c**) The top 10 pathways upregulated in MMD patients compared with healthy controls. (**d**) The top 10 pathways downregulated in MMD patients compared with healthy controls.

Gene ontology (GO) and KEGG pathway analyses were conducted to explore the function of the 2624 differentially expressed mRNAs using DAVID (The Database for Annotation, Visualization and Integrated Discovery). The results showed that upregulated genes were enriched for inflammatory response, response to wounding and defense response, etc. (Fig. 4a), while the downregulated genes were enriched for neurological system process, digestion and positive regulation of transcription from RNA polymerase II promoter, etc. (Fig. 4b). Moreover, the KEGG pathway analysis showed that the upregulated genes were enriched for Toll-like receptor signaling pathway, chemokine signaling pathway and MAPK signaling pathway, etc. (Fig. 4c), while the downregulated genes were enriched for drug metabolism, retinol metabolism and olfactory transduction, etc. (Fig. 4d).

### LncRNA-mRNA co-expression networks

**Figure 5:**
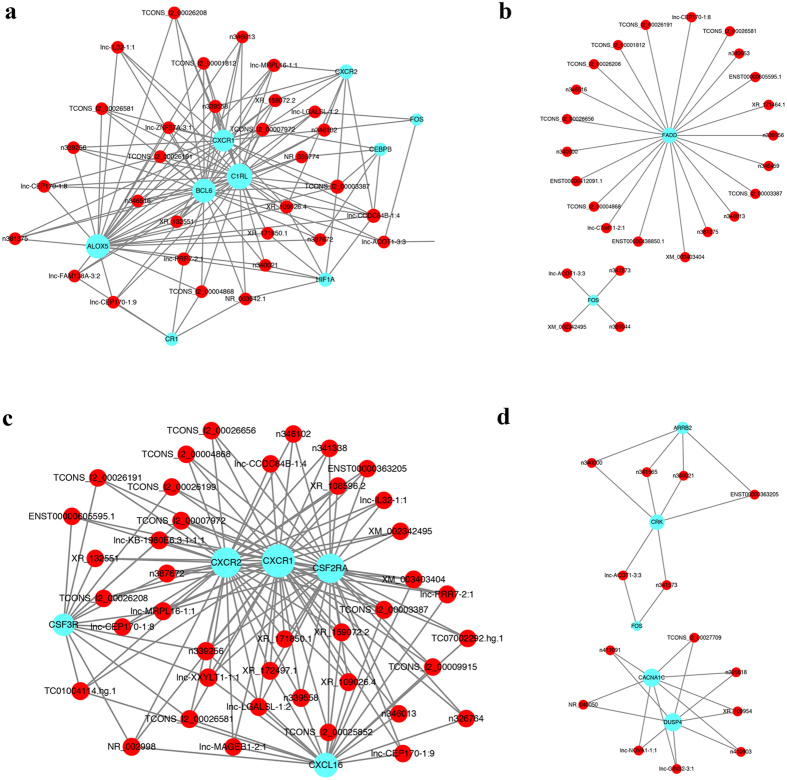
LncRNA-mRNA co-expression network. (**a**) 32 lncRNAs interacted with 11 mRNAs in the GO term of inflammatory response. (**b**) 26 lncRNAs interacted with 2 mRNAs in the Toll-like signaling pathway. (**c**) 41 lncRNAs interacted with 6 mRNAs in the Cytokine-cytokine receptor interaction. (**d**) 15 lncRNAs interacted with 6 mRNAs in the MAPK signaling pathway.

We performed lncRNA-mRNA co-expression network analysis including 3649 lncRNAs probes and 2880 mRNAs probes. Our results showed that the co-expression networks were linked to inflammatory response, Toll-like signaling pathway, cytokine-cytokine receptor interaction and MAPK signaling pathway (Fig. 5). Thirty-two lncRNAs interacted with 11 mRNAs in the GO term of inflammatory response (Fig. 5a), 26 lncRNAs interacted with 2 mRNAs in the Toll-like signaling pathway (Fig. 5b), 41 lncRNAs interacted with 6 mRNAs in the cytokine-cytokine receptor interaction (Fig. 5c), and 15 lncRNAs interacted with 6 mRNAs in the MAPK signaling pathway (Fig. 5d).

## Discussion

MMD is a chronic occlusive cerebrovascular disease of unknown etiology^21^, and it is usually diagnosed by radiological findings, such as computed tomography (CT) perfusion and magnetic resonance imaging (MRI)^22^. It has been shown that MMD has a high prevalence in Asian countries, such as China, Japan and South Korea^23^,^24^,^25^,^26^,^27^. However, there are fewer studies focused on lncRNA-mRNA co-expression in MMD, and the molecular mechanisms behind MMDs remain poorly understood. It has been reported that lncRNAs play important roles in a wide range of functional activities^6^,^7^. The dysregulation of lncRNAs might contribute towards MMD. Therefore, the integrated analysis of the differentially expressed lncRNAs and mRNAs could help to reveal the pathogenesis of MMD.

Many studies have associated single nucleotide polymorphisms (SNPs) of genes with MMD^28^,^29^,^30^,^31^,^32^,^33^,^34^. RNF213 and MMP3 were proposed to be susceptibility genes for MMD^28^,^30^. It was reported that RNF213 is associated with immune response and that it might act cooperatively with other molecules under inflammatory signals based on bioinformatics data. IFNG and TNFA synergistically activated transcription of RNF213 both *in vitro* and *in vivo*^35^. However, IFNG and RNF213 were not co-expressed based on the mRNA microarray. The presence of a heterozygous genotype in TIMP2 promoter could be a genetic factor for familial MMD. Moreover, it was reported that TGFB1 could be involved in vascular growth and transformation processes and may play an important role in the development of MMD^34^,^36^. SNPs may affect the expression of genes for MMD. Based on the microarray database, RNF213 was not differentially expressed between MMDs and controls. However, TGFB1 and TIMP2 were found to be differentially expressed. These findings need to be validated in further studies including more samples.

The co-expression results were based on the expression of lncRNAs and mRNAs for MMD. It was reported that HIF1A could directly bind to the promoter of HOTAIR, which has been identified in a variety of carcinomas^37^. CXCR2 is involved in migration and activation of leukocytes and plays a key role in several inflammatory diseases^38^,^39^. MALAT1 could downregulate the expression of CXCR2 via miR-22–3p^40^. These were consistent with the results that HOTAIR and MALAT1 were highly associated with HIF1A and CXCR2, respectively (correlation coefficient −0.76, 0.87; P < 0.01), based on our microarray. To our knowledge, this is the first study of lncRNA-mRNA co-expression network analysis for patients with MMD. Dai *et al*. have conducted a serum miRNA signature in MMD, and it identified 94 differential expressed miRNAs^41^. Non-coding RNAs and mRNAs compete for binding to miRNAs by sharing one or more miRNA response elements (MREs) to regulate gene expression^42^, and the differentially expressed lncRNAs and mRNAs in our study partly correlate with these miRNAs. Therefore, future studies could focus on finding a competing endogenous RNAs (ceRNA) network with a key role in the pathogenesis of MMDs

Next, we investigated whether mRNAs tend to be neighbors with lncRNAs in MMD. We have performed all lncRNA-mRNA (63431 lncRNAs, 39887 mRNAs) co-expression analyses and lncRNA/mRNA reciprocal expression pattern analyses for MMD patients. Surprisingly, the genomic position and orientation of lncRNAs and mRNAs do have relativity with their correlation coefficient.

We further performed GO and KEGG pathway analyses, which could help us understand the pathogenesis of MMD and could provide new potential therapeutic targets. Moreover, the lncRNA-mRNA co-expression analysis showed that 32 lncRNAs interacted with 11 mRNAs in the GO of inflammatory response and 15 lncRNAs interacted with 6 mRNAs in the MAPK signaling pathway (Fig. 5a,d). Association of differentially expressed lncRNAs and mRNAs with pathways relevant to MMD pathogenesis may partly explain the etiology of MMD. Inflammatory response leads to the hyperplasia of intimal vascular smooth muscle cells (VSMCs), which causes lumen stenosis of MMDs^43^,^44^. The MAPK signaling pathway played important roles in vascular pathological processes^45^,^46^,^47^ and vascular inflammation^48^. It was reported that many cytokines induce the proliferation of VSMCs via MAPK signaling pathway^49^,^50^,^51^. MAPK signaling pathway inhibitors have been successfully used to treat atherosclerosis *in vivo*^52^. Our results can elucidate key lncRNAs and provide leads to further understand the pathogenesis of MMD.

There are limitations to our study. There were only 15 MMD patients and 10 healthy controls included in our analysis, and we could still identify valuable genes and pathways from our database. Further studies would enlarge the sample size. Moreover, the results were obtained from the bioinformatic analysis and microarray analysis. Therefore, further studies are needed to confirm these differentially expressed genes and pathway mechanisms with experiments. This manuscript is our preliminary work, and further work remains to be done.

In conclusion, we identified 880 differentially expressed lncRNAs (3649 probes) and 2624 differentially expressed mRNAs (2880 probes) from the microarray of MMD patients and healthy controls. The upregulated differentially expressed mRNAs were enriched for inflammatory response, the Toll-like receptor signaling pathway, chemokine signaling pathway and the MAPK signaling pathway. The integrated analysis also indicated interregulation in MMD patients. These findings may reveal the pathogenesis of MMD, and future studies should focus on the inflammatory response and MAPK signaling pathway for MMD patients.

## Methods

### Patients and Samples

We enrolled 15 MMD patients who had been diagnosed with MMD according to the characteristic angiographic findings with strict inclusion criteria in Beijing Tiantan Hospital and 10 healthy controls in our study. Informed consent was obtained at enrolment and the basic characteristics of all MMD patients and healthy controls are summarized in Table 1. Our study was approved by the Ethics Committee in Beijing Tiantan hospital. The study was carried out in accordance with the Declaration of Helsinki, and all methods were performed in accordance with the relevant guidelines and regulations.

We obtained the peripheral blood anticoagulated with ethylene diamine tetraacetic acid (EDTA) from all patients and healthy controls. Further, we extracted the RNA by using TRIzol reagent (Invitrogen, Grand Island, NY, USA) according to the manufacturer’s instructions and quality evaluations were performed using Agilent 2100 Bioanalyzer (Agilent Technologies, Santa Clara, CA, USA).

### LncRNA and mRNA Microarrays

The Agilent Human 4 × 180 K lncRNA and mRNA Microarrays (Agilent, Santa Clara, CA, USA) were performed using a Gene Expression Hybridization Kit (Agilent, Santa Clara, CA, US) according to the manufacturer’s instructions. Slides were washed in staining dishes with a Gene Expression Wash Buffer Kit (Agilent, Santa Clara, CA, USA) and scanned by an Agilent Microarray Scanner (Agilent, Santa Clara, CA, USA) with default settings according to the manufacturer’s instructions. Raw data were normalized by Quantile algorithm using Gene Spring Software 12.6 (Agilent Technologies). The differentially expressed lncRNAs and mRNAs were identified by using R software (version 3.2.3) with the samr package^53^.

### Quantitative Real-time Polymerase Chain Reaction (qRT-PCR)

We selected several differentially expressed genes to detect their expression in MMD. Total RNAs were isolated from all samples using PAXgene Blood RNA Kit (Qiagen, Germany) and then reverse transcribed using an iScript cDNA synthesis Kit (Bio-Rad, USA) according to the manufacturer’s instructions. qRT-PCR was performed using SYBR Select Master Mix (Applied Biosystems, USA). Glyceraldehyde 3-phosphate dehydrogenase (GAPDH) was used as an internal control, and all the primer sequences are shown below:

NR_015395-F: 5′-CTAATTTGCCACCACCCTGT-3′

NR_015395-R: 5′-AAGACCCAGATGCCGTTTTA-3′

NR_024420-F: 5′-CTGCAACGAATCCCAAAAGT-3′

NR_024420-R: 5′-ACCACTTTCCAGAGGCTGAA-3′

NR_033908-F: 5′-CGAGCTGTAAAAGCCAAAGG-3′

NR_033908-R: 5′-CCTGGGCGATAAGAGTGAAA-3′

NM_004994-F: 5′-TGTACCGCTATGGTTACACTCG-3′

NM_004994-R: 5′-GGCAGGGACAGTTGCTTCT-3′

NM_005621-F: 5′-ATTGAGGGGTTAACATTAGGCTG-3′

NM_005621-R: 5′-GATATTCTTGATGGTGTTTGCAAGC-3′

GAPDH-F: 5′-AGGGCTGCTTTTAACTCTGGT-3′

GAPDH-R: 5′-CCCCACTTGATTTTGGAGGGA-3′

### Statistical Analysis

All statistical data were analyzed by using SPSS (version 22; SPSS Inc., Chicago, IL, USA) and R software (version 3.2.3). Genes with a two-sided P value of <0.05 and Fold Change >2.0 were regarded as statistically significant genes. The P value was false discovery rate (FDR) corrected. LncRNAs-mRNAs co-expression networks were constructed by Cytoscape software^54^ (version 3.4.0; The Cytoscape Consortium, San Diego, CA, USA).

## Additional Information

**How to cite this article**: Wang, W. *et al*. Integrated Analysis of LncRNA-mRNA Co-Expression Profiles in Patients with Moyamoya Disease. *Sci. Rep.* **7**, 42421; doi: 10.1038/srep42421 (2017).

**Publisher's note:** Springer Nature remains neutral with regard to jurisdictional claims in published maps and institutional affiliations.

## Supplementary Material

Supplementary Information

Supplementary Table S1

## Figures and Tables

**Table 1:** Clinical characteristics of included patients.

Items		MMD	Control
Number		15	10
Sex	Male	8	5
	Female	7	5
Age (Mean ± SD)	Male	32 ± 11.44	31.80 ± 10.30
	Female	32.86 ± 10.57	32.52 ± 10.42
Initial Clinical	IVH	6	ND
	SAH	1	
	TIA	3	
	Infarction	5	
Subgroups	HG	7	ND
	IG	6	
	HIG	2	
mRS Score (Mean ± SD)		1.8 ± 0.68	ND

MMD, Moyamoya disease; IVH, intraventricular hemorrhage; SAH, subarachnoid hemorrhage; TIA, transient ischemic attack; HG, hemorrhagic group; IG, ischemic group; IHG, ischemic and hemorrhagic group; mRs, modified Rankin scale; ND, no data.

## References

[b1] Suzuki J. & Takaku A. Cerebrovascular “moyamoya” disease. Disease showing abnormal net-like vessels in base of brain. Arch Neurol 20, 288–299 (1969). 5775283 10.1001/archneur.1969.00480090076012

[b2] Narisawa A., Fujimura M. & Tominaga T. Efficacy of the revascularization surgery for adult-onset moyamoya disease with the progression of cerebrovascular lesions. Clin Neurol Neurosurg 111, 123–126, doi: 10.1016/j.clineuro.2008.09.022 (2009). 18995956

[b3] Fukui M. Guidelines for the diagnosis and treatment of spontaneous occlusion of the circle of Willis (‘moyamoya’ disease). Research Committee on Spontaneous Occlusion of the Circle of Willis (Moyamoya Disease) of the Ministry of Health and Welfare, Japan. Clin Neurol Neurosurg 99 Suppl 2, S238–240 (1997). 9409446

[b4] Fujimura M. . Genetics and Biomarkers of Moyamoya Disease: Significance of RNF213 as a Susceptibility Gene. J Stroke 16, 65–72, doi: 10.5853/jos.2014.16.2.65 (2014). 24949311 PMC4060268

[b5] Kowalczyk M. S., Higgs D. R. & Gingeras T. R. Molecular biology: RNA discrimination. Nature 482, 310–311, doi: 10.1038/482310a (2012). 22337043

[b6] Mercer T. R., Dinger M. E. & Mattick J. S. Long non-coding RNAs: insights into functions. Nat Rev Genet 10, 155–159, doi: 10.1038/nrg2521 (2009). 19188922

[b7] Fang Y. & Fullwood M. J. Roles, Functions, and Mechanisms of Long Non-coding RNAs in Cancer. Genomics Proteomics Bioinformatics 14, 42–54, doi: 10.1016/j.gpb.2015.09.006 (2016). 26883671 PMC4792843

[b8] Heward J. A. & Lindsay M. A. Long non-coding RNAs in the regulation of the immune response. Trends Immunol 35, 408–419, doi: 10.1016/j.it.2014.07.005 (2014). 25113636 PMC7106471

[b9] Li Z. . The long noncoding RNA THRIL regulates TNFalpha expression through its interaction with hnRNPL. Proc Natl Acad Sci USA 111, 1002–1007, doi: 10.1073/pnas.1313768111 (2014). 24371310 PMC3903238

[b10] Stuhlmuller B. . Detection of oncofetal h19 RNA in rheumatoid arthritis synovial tissue. Am J Pathol 163, 901–911, doi: 10.1016/S0002-9440(10)63450-5 (2003). 12937131 PMC1868271

[b11] Liu Q. . Long noncoding RNA related to cartilage injury promotes chondrocyte extracellular matrix degradation in osteoarthritis. Arthritis Rheumatol 66, 969–978, doi: 10.1002/art.38309 (2014). 24757148

[b12] Pearson M. J. . Long Intergenic Noncoding RNAs Mediate the Human Chondrocyte Inflammatory Response and Are Differentially Expressed in Osteoarthritis Cartilage. Arthritis Rheumatol 68, 845–856, doi: 10.1002/art.39520 (2016). 27023358 PMC4950001

[b13] Su S. . Overexpression of the long noncoding RNA TUG1 protects against cold-induced injury of mouse livers by inhibiting apoptosis and inflammation. FEBS J 283, 1261–1274, doi: 10.1111/febs.13660 (2016). 26785829

[b14] Zhou X. . Long non-coding RNA ANRIL regulates inflammatory responses as a novel component of NF-kappaB pathway. RNA Biol 13, 98–108, doi: 10.1080/15476286.2015.1122164 (2016). 26618242 PMC4829310

[b15] Han Y. . Tumor-suppressive function of long noncoding RNA MALAT1 in glioma cells by downregulation of MMP2 and inactivation of ERK/MAPK signaling. Cell Death Dis 7, e2123, doi: 10.1038/cddis.2015.407 (2016). 26938295 PMC4823926

[b16] Yang H. . Elevated JMJD1A is a novel predictor for prognosis and a potential therapeutic target for gastric cancer. Int J Clin Exp Pathol 8, 11092–11099 (2015). 26617828 PMC4637643

[b17] Bang O. Y., Fujimura M. & Kim S. K. The Pathophysiology of Moyamoya Disease: An Update. J Stroke 18, 12–20, doi: 10.5853/jos.2015.01760 (2016). 26846756 PMC4747070

[b18] Gao F. . Long Noncoding RNAs and Their Regulatory Network: Potential Therapeutic Targets for Adult Moyamoya Disease. World Neurosurgery 93, 111–119, doi: 10.1016/j.wneu.2016.05.081 (2016). 27268316

[b19] Xu J. . Differentially expressed lncRNAs and mRNAs identified by microarray analysis in GBS patients vs healthy controls. Sci Rep 6, 21819, doi: 10.1038/srep21819 (2016). 26898505 PMC4761882

[b20] Xu J. . Microarray Analysis of lncRNA and mRNA Expression Profiles in Patients with Neuromyelitis Optica. Mol Neurobiol, doi: 10.1007/s12035-016-9754-0 (2016). PMC5355516 26941100

[b21] Kim J. S. Moyamoya Disease: Epidemiology, Clinical Features, and Diagnosis. J Stroke 18, 2–11, doi: 10.5853/jos.2015.01627 (2016). 26846755 PMC4747069

[b22] Kuroda S. & Houkin K. Moyamoya disease: current concepts and future perspectives. Lancet Neurol 7, 1056–1066, doi: 10.1016/S1474-4422(08)70240-0 (2008). 18940695

[b23] Kuriyama S. . Prevalence and clinicoepidemiological features of moyamoya disease in Japan: findings from a nationwide epidemiological survey. Stroke 39, 42–47, doi: 10.1161/STROKEAHA.107.490714 (2008). 18048855

[b24] Han D. H. . A co-operative study: clinical characteristics of 334 Korean patients with moyamoya disease treated at neurosurgical institutes (1976–1994). The Korean Society for Cerebrovascular Disease. *Acta Neurochir (Wien*) 142, 1263–1273; discussion 1273–1264 (2000). 10.1007/s007010070024 11201642

[b25] Hung C. C., Tu Y. K., Su C. F., Lin L. S. & Shih C. J. Epidemiological study of moyamoya disease in Taiwan. Clin Neurol Neurosurg 99 Suppl 2, S23–25 (1997). 9409399 10.1016/s0303-8467(97)00036-x

[b26] Miao W. . Epidemiological and clinical features of Moyamoya disease in Nanjing, China. Clin Neurol Neurosurg 112, 199–203, doi: 10.1016/j.clineuro.2009.11.009 (2010). 20004511

[b27] Duan L. . Moyamoya disease in China: its clinical features and outcomes. Stroke 43, 56–60, doi: 10.1161/STROKEAHA.111.621300 (2012). 22020027

[b28] Kamada F. . A genome-wide association study identifies RNF213 as the first Moyamoya disease gene. J Hum Genet 56, 34–40, doi: 10.1038/jhg.2010.132 (2011). 21048783

[b29] Kang H. S. . Single nucleotide polymorphisms of tissue inhibitor of metalloproteinase genes in familial moyamoya disease. Neurosurgery 58, 1074–1080; discussion 1074–1080, doi: 10.1227/01.NEU.0000215854.66011.4F (2006). 16723886

[b30] Li H. . Association of a functional polymorphism in the MMP-3 gene with Moyamoya Disease in the Chinese Han population. Cerebrovasc Dis 30, 618–625, doi: 10.1159/000319893 (2010). 20948207

[b31] Liu C. . Analysis of TGFB1 in European and Japanese Moyamoya disease patients. Eur J Med Genet 55, 531–534, doi: 10.1016/j.ejmg.2012.05.002 (2012). 22659181

[b32] Liu W. . A rare Asian founder polymorphism of Raptor may explain the high prevalence of Moyamoya disease among East Asians and its low prevalence among Caucasians. Environ Health Prev Med 15, 94–104, doi: 10.1007/s12199-009-0116-7 (2010). 19921495 PMC2824103

[b33] Park Y. S. . Age-specific eNOS polymorphisms in moyamoya disease. Childs Nerv Syst 27, 1919–1926, doi: 10.1007/s00381-011-1504-z (2011). 21691823

[b34] Roder C. . Polymorphisms in TGFB1 and PDGFRB are associated with Moyamoya disease in European patients. Acta Neurochir (Wien) 152, 2153–2160, doi: 10.1007/s00701-010-0711-9 (2010). 20571834

[b35] Ohkubo K. . Moyamoya disease susceptibility gene RNF213 links inflammatory and angiogenic signals in endothelial cells. Sci Rep 5, 13191, doi: 10.1038/srep13191 (2015). 26278786 PMC4538604

[b36] Hojo M. . Role of transforming growth factor-beta1 in the pathogenesis of moyamoya disease. J Neurosurg 89, 623–629, doi: 10.3171/jns.1998.89.4.0623 (1998). 9761057

[b37] Zhou C. . Long noncoding RNA HOTAIR, a hypoxia-inducible factor-1alpha activated driver of malignancy, enhances hypoxic cancer cell proliferation, migration, and invasion in non-small cell lung cancer. Tumour Biol 36, 9179–9188, doi: 10.1007/s13277-015-3453-8 (2015). 26088446

[b38] Bizzarri C. . ELR + CXC chemokines and their receptors (CXC chemokine receptor 1 and CXC chemokine receptor 2) as new therapeutic targets. Pharmacol Ther 112, 139–149, doi: 10.1016/j.pharmthera.2006.04.002 (2006). 16720046

[b39] Muller-Edenborn B. . Volatile anaesthetics reduce neutrophil inflammatory response by interfering with CXC receptor-2 signalling. Br J Anaesth 114, 143–149, doi: 10.1093/bja/aeu189 (2015). 24989774

[b40] Tang Y. . The lncRNA MALAT1 protects the endothelium against ox-LDL-induced dysfunction via upregulating the expression of the miR-22-3p target genes CXCR2 and AKT. FEBS Lett 589, 3189–3196, doi: 10.1016/j.febslet.2015.08.046 (2015). 26364720

[b41] Dai D. . Serum miRNA signature in Moyamoya disease. PLoS One 9, e102382, doi: 10.1371/journal.pone.0102382 (2014). 25093848 PMC4122349

[b42] Salmena L., Poliseno L., Tay Y., Kats L. & Pandolfi P. P. A ceRNA hypothesis: the Rosetta Stone of a hidden RNA language? Cell 146, 353–358, doi: 10.1016/j.cell.2011.07.014 (2011). 21802130 PMC3235919

[b43] Takekawa Y., Umezawa T., Ueno Y., Sawada T. & Kobayashi M. Pathological and immunohistochemical findings of an autopsy case of adult moyamoya disease. Neuropathology 24, 236–242 (2004). 15484702 10.1111/j.1440-1789.2004.00550.x

[b44] Weinberg D. G. . Moyamoya disease: a review of histopathology, biochemistry, and genetics. Neurosurg Focus 30, E20, doi: 10.3171/2011.3.FOCUS1151 (2011). 21631222

[b45] Park K. M., Chen A. & Bonventre J. V. Prevention of kidney ischemia/reperfusion-induced functional injury and JNK, p38, and MAPK kinase activation by remote ischemic pretreatment. J Biol Chem 276, 11870–11876, doi: 10.1074/jbc.M007518200 (2001). 11150293

[b46] Nordmeyer J. . Upregulation of myocardial estrogen receptors in human aortic stenosis. Circulation 110, 3270–3275, doi: 10.1161/01.CIR.0000147610.41984.E8 (2004). 15533858

[b47] Hu Y. . Icariin Attenuates High-cholesterol Diet Induced Atherosclerosis in Rats by Inhibition of Inflammatory Response and p38 MAPK Signaling Pathway. Inflammation 39, 228–236, doi: 10.1007/s10753-015-0242-x (2016). 26307750

[b48] Paudel K. R., Karki R. & Kim D. W. Cepharanthine inhibits *in vitro* VSMC proliferation and migration and vascular inflammatory responses mediated by RAW264.7. Toxicol In Vitro 34, 16–25, doi: 10.1016/j.tiv.2016.03.010 (2016). 27021874

[b49] Cui Y. . Platelet-derived growth factor-BB induces matrix metalloproteinase-2 expression and rat vascular smooth muscle cell migration via ROCK and ERK/p38 MAPK pathways. Mol Cell Biochem 393, 255–263, doi: 10.1007/s11010-014-2068-5 (2014). 24792035

[b50] Suwanabol P. A. . Transforming growth factor-beta increases vascular smooth muscle cell proliferation through the Smad3 and extracellular signal-regulated kinase mitogen-activated protein kinases pathways. J Vasc Surg 56, 446–454, doi: 10.1016/j.jvs.2011.12.038 (2012). 22521802 PMC3408812

[b51] Martin-Garrido A. . Transforming growth factor beta inhibits platelet derived growth factor-induced vascular smooth muscle cell proliferation via Akt-independent, Smad-mediated cyclin D1 downregulation. PLoS One 8, e79657, doi: 10.1371/journal.pone.0079657 (2013). 24236150 PMC3827379

[b52] Zhao M. . Activation of the p38 MAP kinase pathway is required for foam cell formation from macrophages exposed to oxidized LDL. APMIS 110, 458–468 (2002). 12193207 10.1034/j.1600-0463.2002.100604.x

[b53] Huang X., Hao C., Bao H., Wang M. & Dai H. Aberrant expression of long noncoding RNAs in cumulus cells isolated from PCOS patients. J Assist Reprod Genet 33, 111–121, doi: 10.1007/s10815-015-0630-z (2016). 26650608 PMC4717141

[b54] Shannon P. . Cytoscape: a software environment for integrated models of biomolecular interaction networks. Genome Res 13, 2498–2504, doi: 10.1101/gr.1239303 (2003). 14597658 PMC403769

