# Supplementary material for: Integrated Analysis of LncRNA-mRNA Co-Expression Profiles in Patients with Moyamoya Disease
Source: Sci Rep. 2017 Feb 8;7:42421. doi: 10.1038/srep42421 (PMC5296735; doi:10.1038/srep42421)
Supplement: Supplementary Information [file srep42421-s1.pdf]

Integrated Analysis of LncRNA-mRNA Co-Expression Profiles in Patients with Moyamoya Disease

Wen Wang[1,2,5,6,*], Faliang Gao[1,5,6,*], Zheng Zhao[3], Haoyuan Wang[4], Lu Zhang[7], Dong Zhang[1,5,6], Yan Zhang[1,5,6], Qing Lan[2], Jiangfei Wang[1,5,6], Jizong Zhao[1,2,5,6]

1. Department of Neurosurgery, Beijing Tiantan Hospital, Capital Medical University, Beijing, China, 100050

2. Department of Neurosurgery, The Second Affiliated Hospital of Soochow University, Suzhou, China, 215123

3. Beijing Neurosurgical Institute, Capital Medical University, Beijing, China, 100050

4. Department of Neurosurgery, Zhujiang Hospital, Southern Medical University, Guangzhou, China, 510280

5. China National Clinical Research Center for Neurological Diseases, Beijing, China, 100050

6. Beijing Key Laboratory of Translational Medicine for Cerebrovascular Diseases, Beijing, China, 100050

7. Department of Ophthalmology, School of Medicine, Shandong University, Jinan, China, 250012

*These authors contributed equally to this work.

Corresponding author: Jizong Zhao, Department of Neurosurgery, Beijing Tiantan Hospital, Capital Medical University, No. 6 Tiantan Xili, Dongcheng District, Beijing, China, 100050. Tele: +86-18678522377; Fax: 010-67096611. E-mail: zhaojz205@163.com

Supplementary Information

Table S1. The identified differentially expressed lncRNAs and mRNAs of MMDs.
